# A new safety index based on intrapulse monitoring of ultra-harmonic cavitation during ultrasound-induced blood-brain barrier opening procedures

**DOI:** 10.1038/s41598-020-66994-8

**Published:** 2020-06-22

**Authors:** A. Novell, H. A. S. Kamimura, A. Cafarelli, M. Gerstenmayer, J. Flament, J. Valette, P. Agou, A. Conti, E. Selingue, R. Aron Badin, P. Hantraye, B. Larrat

**Affiliations:** 1 Université Paris-Saclay, CEA, CNRS, Baobab, NeuroSpin, Gif-sur-Yvette, 91191 France; 2 Université Paris-Saclay, CEA, CNRS, Inserm, BioMaps, Orsay, 91401 France; 3 Université Paris-Saclay, CEA, CNRS, Neurodegenerative Diseases Laboratory, Molecular Imaging Research Center (MIRCen), Fontenay-aux-Roses, 92265 France; 4 Present Address: Department of Biomedical Engineering, Columbia University, New York, NY USA; 5 The BioRobotics Institute, Scuola Superiore Sant’Anna, Pisa, 56025 Italy

**Keywords:** Drug delivery, Biomedical engineering

## Abstract

Ultrasound-induced blood-brain barrier (BBB) opening using microbubbles is a promising technique for local delivery of therapeutic molecules into the brain. The real-time control of the ultrasound dose delivered through the skull is necessary as the range of pressure for efficient and safe BBB opening is very narrow. Passive cavitation detection (PCD) is a method proposed to monitor the microbubble activity during ultrasound exposure. However, there is still no consensus on a reliable safety indicator able to predict potential damage in the brain. Current approaches for the control of the beam intensity based on PCD employ a full-pulse analysis and may suffer from a lack of sensitivity and poor reaction time. To overcome these limitations, we propose an intra-pulse analysis to monitor the evolution of the frequency content during ultrasound bursts. We hypothesized that the destabilization of microbubbles exposed to a critical level of ultrasound would result in the instantaneous generation of subharmonic and ultra-harmonic components. This specific signature was exploited to define a new sensitive indicator of the safety of the ultrasound protocol. The approach was validated *in vivo* in rats and non-human primates using a retrospective analysis. Our results demonstrate that intra-pulse monitoring was able to exhibit a sudden appearance of ultra-harmonics during the ultrasound excitation pulse. The repeated detection of such a signature within the excitation pulse was highly correlated with the occurrence of side effects such as hemorrhage and edema. Keeping the acoustic pressure at levels where no such sign of microbubble destabilization occurred resulted in safe BBB openings, as shown by MR images and gross pathology. This new indicator should be more sensitive than conventional full-pulse analysis and can be used to distinguish between potentially harmful and safe ultrasound conditions in the brain with very short reaction time.

## Introduction

The blood-brain barrier (BBB) regulates cerebral homeostasis and prevents the passage of xenobiotics and pathogens from the vasculature into the central nervous system (CNS). Due to the selective permeability of the BBB, the majority of drugs cannot reach the brain parenchyma at therapeutic concentrations^1^. Transcranial focused ultrasound (FUS) can induce cavitation, which may induce transient, localized BBB disruption to allow the passage of molecules into the brain^2^. Promising results have been shown for the treatment of brain diseases and other conditions using this technique. Nevertheless, fine control of acoustic parameters is crucial to avoid excessive microbubble activity that could result in vascular damage^3,4^. Thus, work along these lines is necessary for (i) the definition of a reliable monitoring readout during the procedure for safe and efficient ultrasound-assisted BBB disruption; and (ii) a better understanding of biophysical phenomena involved in this process.

Microbubble cavitation can be controlled by tuning ultrasound parameters (*e.g*., pressure amplitude, frequency, pulse repetition frequency, burst length). Low acoustic pressures induce stable cavitation (alternation of expansion and shrinkage of microbubbles) near the vessel wall that can result in tight junction loosening due to local stress via push-pull mechanisms or microstreaming^4,5^. On the other hand, higher ultrasound intensities may induce inertial cavitation that leads to violent collapse and fragmentation of microbubbles accompanied by micro-jets and shock waves. It is generally accepted that inertial cavitation should be avoided as it is not required for successful BBB opening and may be associated with the presence of undesirable vessel damage^3,4,6^. Recording and using cavitation signals during the BBB opening is now widely accepted as a very efficient way to control the procedure in real-time and to ensure repeatable, efficient, and safe delivery to the brain. However, there is still no consensus on the best method to do so.

Commercial ultrasound contrast agents currently available for BBB opening in humans consist of lipid-stabilized microbubbles (*e.g*., Definity, Lantheus Medical Imaging; SonoVue, Bracco Imaging). The time for microbubble distribution and the intensity of microbubble oscillation induced by ultrasound are directly related to their therapeutic efficacy and safety^7^. Ultrasound waves set circulating microbubbles into nonlinear oscillations that generate specific harmonic components (*e.g*., subharmonic, harmonic, ultra-harmonic)^8^. Conversely, at higher pressures, inertial cavitation collapses microbubbles, generating broadband emissions. The occurrence of subharmonic and ultra-harmonic frequencies is typically interpreted as a threshold for inertial cavitation that is associated with a risky oscillation regime of microbubbles^9^. These unique acoustic signatures provide monitoring information about microbubble activity through passive cavitation detection (PCD)^6,10,11^. Feedback-control algorithms based on the detection of inertial cavitation^12,13^ have yielded safe BBB disruption. However, the tolerable cavitation threshold allowing efficient opening remains challenging to determine. Hence, other approaches based on the monitoring of specific frequency components such as harmonic^14^, subharmonic^15^, and ultra-harmonic^10,14,16^ have also been proposed as indicators of efficient and safe BBB opening.

Overall, there is still no consensus on the meaning of the presence of subharmonic and ultra-harmonic frequency components or whether these could reliably indicate harmful microbubble activity or not. O’Reilly and Hynynen have proposed a real-time feedback-controlled BBB-disruption based on the modulation of acoustic pressures^10^. The approach consisted of reducing the pressure amplitude once ultra-harmonic emissions were detected. The level of ultra-harmonic emission was calculated after each 10-ms burst and compared with the reference value before microbubble injection. On the contrary, Bing *et al*. defined a feedback control algorithm to maintain a high level of acoustic emissions (sub and ultra-harmonics) throughout the entire treatment by adjusting the pressure amplitude^17^. Although the results indicated successful BBB-disruption using different contrast agents, no safety study assessed potential damage to surrounding tissues.

Some studies reported the difficulty of isolating both sub- and ultra-harmonic from broadband emissions that indicated inertial cavitation^13,14^ and concluded that these specific signatures were not useful for predicting BBB disruption or damage. However, in all these studies, cavitation was calculated by averaging the frequency response on the total burst length (few ms). As a result, many considerations are necessary for the analysis of the ultrasound-induced microbubble activity and to conclude on its influence on the resulting BBB opening^18,19^.

Shekhar *et al*. observed *in vitro* an increase in both subharmonic and ultra-harmonic responses 5–10 min after lipid-shelled microbubble preparation^20^. The time evolution of these frequency components is attributed to mechanical changes in microbubble shape and behavior due to gas-exchange through its shell. Interestingly, other frequency components (*i.e*., fundamental and harmonic response) remained unchanged. Thus, the temporal evolution of subharmonic and ultra-harmonic responses can be considered a specific marker of changes in microbubble formulation and properties (*i.e*., size, gas composition, and shell properties). Also, ultrasound exposure may alter the properties of the microbubble shells and accelerate its destabilization, causing shell buckling, lipid shedding, microbubble deflation, dissolution, and fragmentation^21–25^. In particular, shell buckling has been possibly associated with the occurrence of subharmonic oscillations;^26,27^ shell degradation has been linked with the evolution of specific microbubble spectral signature^28^; and microbubble deflation has been attributed to gas diffusion also affecting the nonlinear response of microbubbles^29^. It has been reported that the use of higher acoustic pressure (typically peak negative pressure above 300 kPa) and long bursts (several dozens of cycles) increases the occurrence of shedding events^22^ or coalescence^25^ of lipid-shelled microbubbles. All these phenomena may result in microbubble destabilization leading to their destruction and could potentially increase the risk of inducing harmful biological effects. Thus, these characteristics could be better explored in feedback controllers.

In this study, we monitored the evolution of ultra-harmonic frequency components throughout the ultrasound burst during *in vivo* ultrasound-mediated BBB opening sessions in rats and non-human primates (NHP). We propose a new intra-pulse analysis that allows the detection of these specific frequency components during the ultrasound bursts. By doing so, we enable very short reaction time for future feedback controllers. The acquisition of baseline signals before microbubbles are injected, a step that is both time consuming and sensitive to motion or transducer coupling artifacts, is not needed anymore (12). Intra-pulse processing also eliminates the averaging process over the full-pulse that could decrease the sensitivity to detect sudden changes in subharmonic and ultra-harmonic emissions. Finally, we interpreted the relation of these specific frequency components with the safety and efficacy of BBB opening.

## Methods

### Ultrasound protocols for in vivo experiments

All experiments were conducted following European (EU Directive 2010/63), French regulations (French Act Rural Code R 214-87 to 126), and performed in compliance with Standards for Humane Care and Use of Laboratory Animals of the Office of Laboratory Animal Welfare (OLAW – n°#A5826-01). The experimental protocol was approved by a local ethics committee (Comité d'Éthique en Expérimentation Animale du Commissariat à l'Énergie Atomique et aux énergies alternatives Direction des Sciences du Vivant Ile de France, CEtEA n°44) and by the French Ministry of Research and Education (NHP: Authorization n° APAFIS#908-2015062410594279v2; rat: project authorization number: 12-058, site authorization number: B-91-272-01). All efforts were made to minimize animal suffering, and animal care was supervised by veterinarians and animal technicians skilled in the healthcare and housing rodents and NHPs. Body temperature and respiratory rate were monitored throughout the experiments. All animal heads were shaved and covered with degassed acoustic coupling gel.

### Macaque protocol

NHP experiments were conducted in three male cynomolgus monkeys (Macaca fascicularis, age: four to six years, weight: 4.1–7.9 kg, supplied by Noveprim, Mauritius Island), sedated with a mixture of ketamine/xylazine (10:1 mg/kg) and maintained by an intravenous infusion of propofol (1 ml/kg/h). The animals were placed in a stereotaxic frame in a supine position and covered with a flexible heating pad (SA Instruments Inc., NY, USA) that maintained the animal’s body temperature at 37 °C.

The ultrasound setup used for NHP experiments^12^ consisted of an MR-compatible annular array ultrasonic transducer (center frequency: 500 kHz, number of elements: 14, spherical focusing radius: 6 cm; Imasonic SAS, Voray sur l’Ognon, France) designed with spherical shape and a hole in the center for the placement of another ultrasonic transducer used for PCD (center frequency: 1.53 MHz, frequency bandwidth: 58%, diameter: 4.5 mm; Imasonic, Voray sur l’Ognon, France). The transducer was driven by a multi-channel programmable RF-amplifier (LabFUS, Image Guided Therapy, Pessac, France). After intravenous injection of lipid-coated microbubbles (dose: 0.30 mL/kg; SonoVue, Bracco Imaging S.p.A., Milan, Italy), local sonication was applied for a maximum of 2 min with peak negative pressure ranging from 0.09 to 1.16 MPa (calibrated in free water), 500-kHz frequency, the pulse repetition frequency of 5 Hz or 10 Hz and pulse length of 10 ms.

For ethical reasons, macaques were not sacrificed at the end of the BBB opening session, and some of them participated in multiple sessions of BBB opening in different brain locations (*i.e*., 15 sessions in total).

### Rat protocol

The experiments on rodents included a total of 8 Sprague Dawley rats (male and female with 175 to 560 g; Janvier, Le Genest-Saint-Isle, France). Rats were anesthetized with 1.5% isoflurane in a mixture (50:50) of air and oxygen (n = 3) or a cocktail of Xylazine/Ketamine (10 mL/kg intraperitoneal; 10 mg/kg Xylazine; 100 mg/kg Ketamine) (n = 5), and placed in a cradle in prone position. A catheter (25 G needle) was positioned in the caudal vein to inject microbubbles and Evans Blue (used to reveal BBB disruption) from outside the MRI scanner. One percent heparin was added to all injected solutions to avoid clot formation in the catheter.

For rat experiments, ultrasound was applied by an MR-compatible focused annular array transducer (center frequency 0.65 MHz, number of elements: 6, diameter 30 mm, focal depth 30 mm; Imasonic SAS, Voray sur l’Ognon, France) driven by a programmable ultrasound multi-channel amplifier (LabFUS, Image Guided Therapy, Pessac, France). An unfocused transducer (center frequency: 2 MHz, diameter: 2 mm; Imasonic SAS, Voray sur l’Ognon, France) was used in receive mode to record microbubble cavitation. After positioning the transducer over the rat head, microbubbles (200 µL; SonoVue, Bracco Imaging S.p.A., Milan, Italy) were injected in the tail vein, and sonication was applied for 2 min with peak negative pressure ranging from 0.09 to 1.02 MPa (calibrated in free water), 650-kHz frequency, the pulse repetition frequency of 5 Hz and pulse length of 7.7 ms. Each rat underwent a single BBB opening session (*i.e*., eight sessions in total) and was euthanized at the end of the experiment for post-mortem safety assessment.

### Cavitation signal processing

For all experiments, PCD was performed during ultrasound exposure for real-time monitoring of microbubble activity. The signal detected by the PCD transducer was acquired (sampling frequency: 15.625 MHz or 31.25 MHz) by an oscilloscope (PicoScope 5242B, Pico Technology, Cambridgeshire, UK), and transferred to a computer using a signal processing software developed in Python (version 2.7.12, Python Software Foundation, Delaware, USA) for signal processing. The ultrasound system triggered the acquisition.

**Figure 1:**
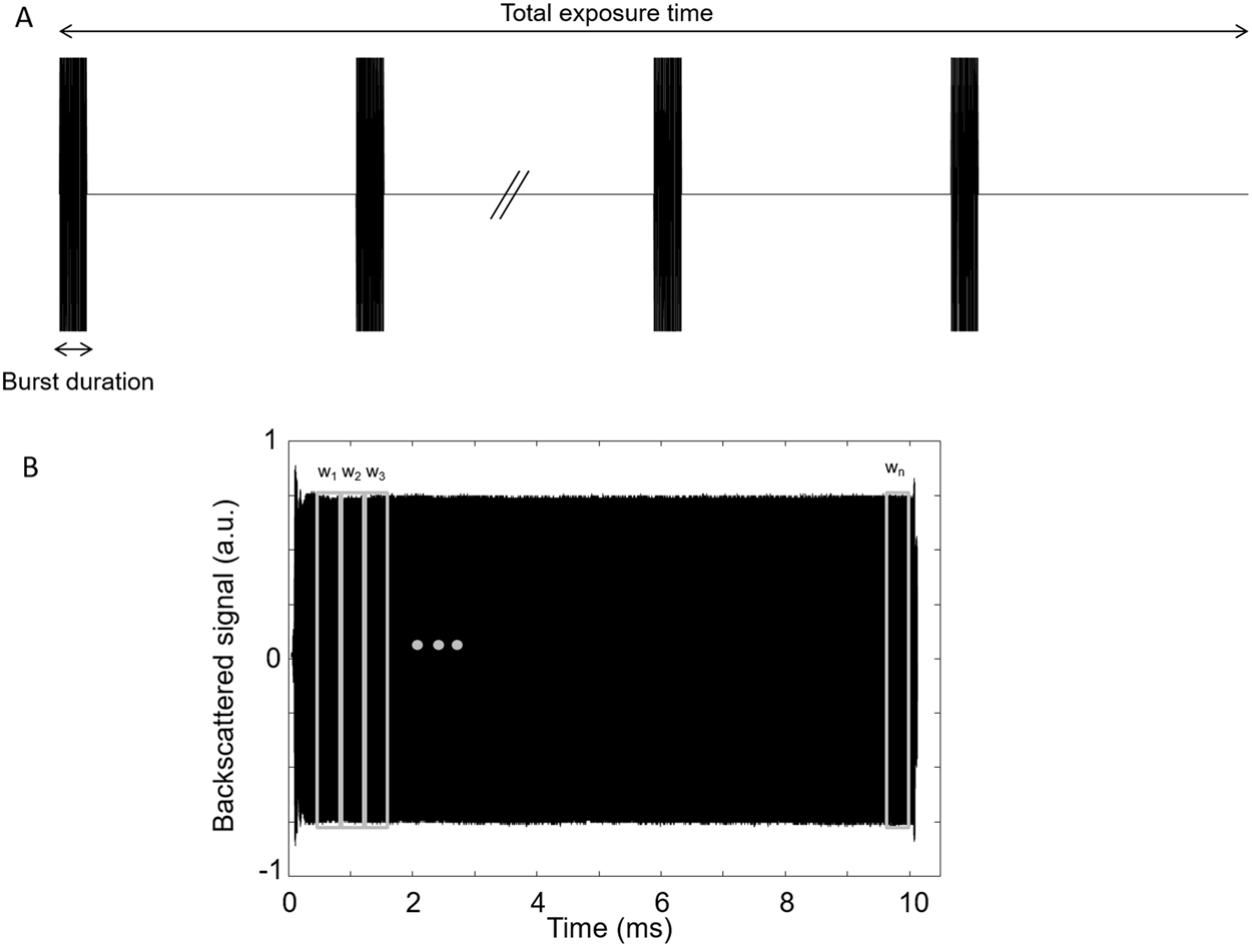
(**A**) Representation of a typical ultrasound sequence for BBB opening. In general, multiple bursts of 3–10 ms are repeated at a pulse repetition frequency of 5–10 Hz, for a total exposure time ranging from 0.5 to 5 minutes. (**B**) The ultrasound backscattered signal originated from the cavitation of microbubbles is decomposed into *n* temporal windows for comparative frequency analysis.

A traditional ultrasound sequence designed for BBB opening is usually composed of multiple bursts, as shown in Fig. 1A. For each ultrasound burst, the frequency response of the backscattered signal is calculated in real-time, and its frequency content is used to determine the cavitation doses (*i.e*., stable or inertial) and can be used to adjust the beam intensity. In practice, the frequency response is averaged over the total length of an echo (*i.e*., the length of the corresponding burst excitation). In this study, we propose a new safety index named intrapulse ultra-harmonic dose (IUD), defined as the relative amplitude in the intrapulse evolution of ultra-harmonic emissions. The raw PCD data is processed in the following way for the calculation of the IUD:

1. Step 1: The echo measured for each ultrasound burst is segmented in *n* temporal windows (*w*_*n*_) of equivalent length to assess the evolution of the frequency content inside the burst. The number of windows (*n*) and their length (*t*_*w*_) is directly dependent on the length and the frequency of the burst defined in the ultrasound sequence. An initial delay was considered before defining the 1^st^ window (indicated in Table 1 as “initial pulse cut”) to avoid significant amplitude variations that could occur at the beginning of the signal (amplifier rise time, transducer response, impedance change due to reflections). This delay allowed the burst to reach a steady-state and to make it possible to compare a series of temporal windows. This initial delay might be adjusted depending on the transducer, electronics, and setup used. The windowing was also halted before the end of the echo for the same reason.

**Table 1:** Summary of the ultrasound emission and reception parameters used for NHP and rat experiments.

	Macaques	Rats
Emission central frequency	0.50 MHz	0.65 MHz
Reception central frequency	1.53 MHz	2.00 MHz
Sonication scheme	Single spot	Single spot
Pulse duration / Pulse Repetition Frequency	10 ms / 5 Hz or 10 Hz	7.7 ms / 5 Hz
PNP range (in water)	0.09–1.16 MPa	0.09–1.02 MPa
Maximum sonication duration	2 min	2 min
PCD sampling rate	15.625 MHz or 31.25 MHz	15.625 MHz or 31.25 MHz
Analyzed ultra-harmonic components	1.5, 2.5 and 3.5 f0	1.5, 2.5 and 3.5 f_0_
Initial pulse cut	From 288 µs to 320 µs	192 µs
Number of time windows / pulse	75	55
Duration of elementary windows	128 µs	127.7 µs
2. Step 2: For each temporal window (*w*_*k*_, 1 < k < *n*), Fast Fourier Transform was performed to convert the data in the frequency domain.
3. Step 3: For each temporal window (*w*_*k*_, 1 < k < *n*), the cavitation signal *s*_*k*_ was determined from the frequency response by calculating the areas under the curve (AUC) corresponding to the multiple ultra-harmonic components (1.5 f_0_, 2.5 f_0_, and 3.5 f_0_). The cavitation signal was calculated by summing the contribution of those components as follows: ${s}_{k}={{AUC}}_{\frac{3f0}{2}}(k)+{{AUC}}_{\frac{5f0}{2}}(k)+{{AUC}}_{\frac{7f0}{2}}(k)$, where the contribution of the subharmonic component (0.5 f_0_) could be added. However, the subharmonic component was not considered in our post-processing due to the lack of sensitivity of our PCD transducer at 0.5 f_0_ (microbubble activity was monitored into the brain using a PCD transducer centered at three times the excitation frequency f_0_).
4. Step 4: To calculate the IUD of a temporal window *w*_*k*_, the cavitation signal *s*_*k*_ was normalized by the cavitation signal *s*_1_ from the 1^st^ temporal window (corresponding to the initial state of interrogated microbubbles, at the beginning of the excitation) such as IUD_k_ = $\frac{{s}_{k}}{{s}_{1}}$. IUD can be expressed as a linear factor or in dB. A microbubble destabilization event was recorded every time the IUD exceeded a threshold value (8 dB in this study for NHP and rat experiments).

For NHP, each backscattered signal was segmented in 75 windows of 128 µs. IUD was calculated for ultra-harmonics centered at *α f*_0_ (where *α* = 1.5, 2.5 or 3.5 and *f*_0_ = 500 kHz) considering a lobe width *Δf* of 20 kHz (*α f*_0_ ± 10 kHz). Similarly, 55 windows of 127.7 µs were considered for rats. IUD was calculated for ultra-harmonics centered at *α f*_0_ (where *α* = 1.5, 2.5 or 3.5 and *f*_0_ = 650 kHz) with *Δf* = 4 kHz.

### PCD-based feedback control

The feedback control described in^12^ was applied for NHP and rat experiments. Briefly, the sonication started at half the maximum tolerated pressure (*i.e.*, 580 kPa for NHP, 501 kPa for rats) and increased gradually with 9 kPa steps until a defined stable cavitation index was reached or an inertial cavitation event was detected . The stable cavitation dose (SCD) was determined as the sum of the root mean square (RMS) of the harmonic and ultra-harmonic frequency components. The inertial cavitation dose (ICD) was determined as the RMS of the broadband signal, excluding the frequency bandwidths of the SCD.

### BBB opening assessment and safety follow up by MRI

Both NHP and rat experiments were performed in 7 T MRI scanners equipped with dedicated MR compatible cradles^12,30^. The MRI guidance enabled to check the acoustic coupling (*i.e.*, bubbles trapped in the gel) and target planning. The shielded MRI environment was beneficial for reducing noise on PCD recordings during sonication. Moreover, MRI was used to verify the BBB opening and assess the brain tissue and vasculature after sonication. The imaging protocol for NHP (same as in (12)) consisted of monitoring the gadolinium chelate uptake in the brain parenchyma through T_1_-weighted dynamic contrast-enhanced images acquired immediately after sonication. T_2_*-weighted images assessed safety by detecting iron deposits due to bleedings. For rat experiments, brain gross pathology using Evans blue dye was used to evaluate the safety and efficacy of the BBB disruption protocol in addition to MRI. T_2_-weighted images were acquired at the end of the sonication to detect the potential presence of hemorrhages or edema at the brain sonicated region. A Rapid Acquisition with Refocused Echoes (RARE) sequence with the following parameters was used: TR = 3000 ms, TE = 34.2 ms, 125 × 125 mm^2^ in-plane resolution, 10 coronal slices, slice thickness = 1 mm, RARE factor = 8, 6 averages, acquisition time = 9.36 min. BBB disruption was assessed by intravenous administration of Evans Blue (dose: 1.5 mL/kg) just before the sonication procedure.

## Results

### Detection of ultra-harmonic events on non-human primate BBB opening

A total of 15 BBB-opening sessions in macaques have been re-processed or newly performed in this paper. Four of these sessions were executed without feedback control sonication and eleven with the feedback control. Based on MRI follow up, only one session resulted in an evident brain hemorrhage and the second one in a suspicious hemorrhage case.

**Figure 2:**
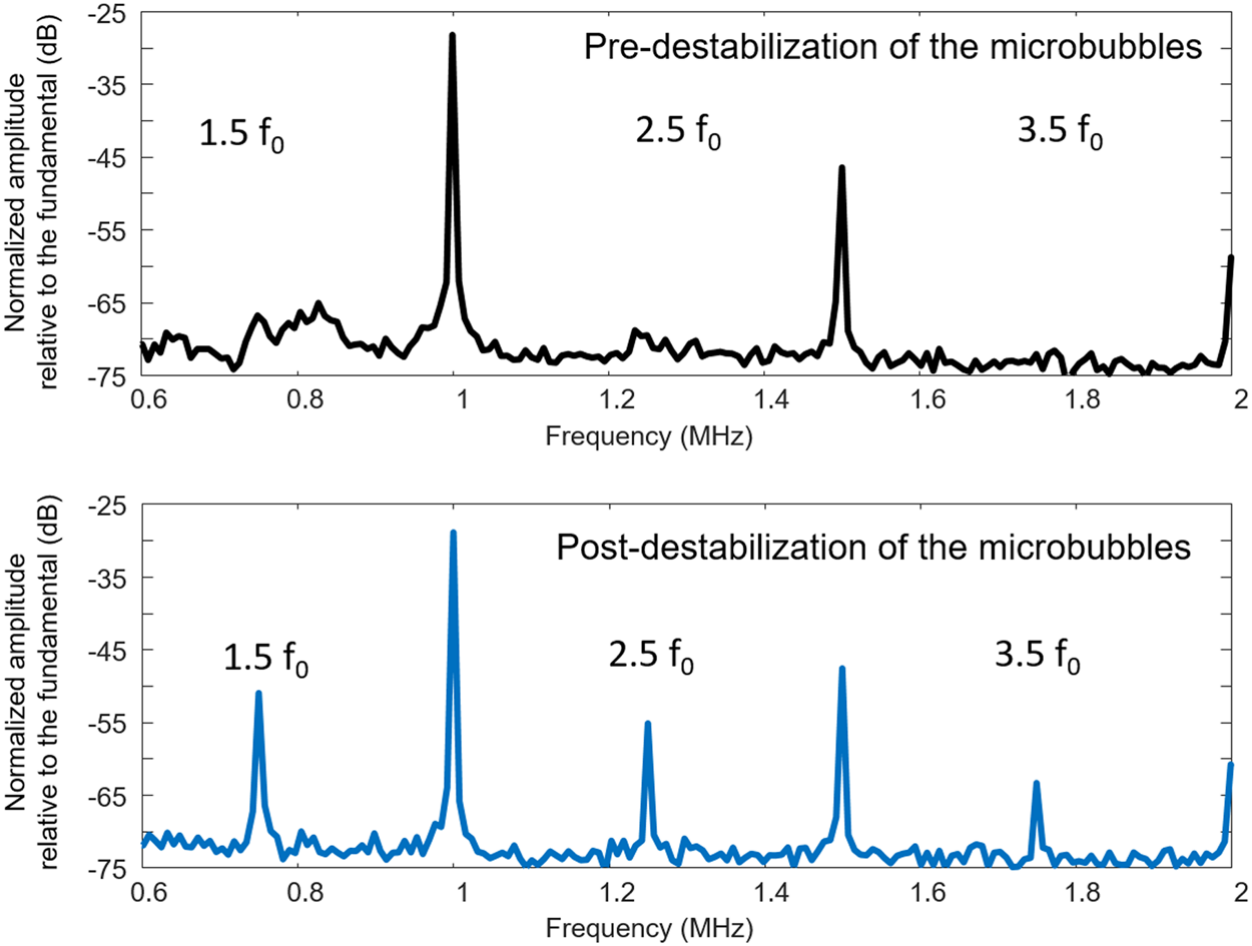
Representative frequency responses of a microbubble cavitation signal measured at different periods (before and after 3.3 ms) following ultrasound bursts. Transcranial ultrasound was emitted at 0.5 MHz into the NHP brain, and the cavitation signal was measured using a PCD transducer centered at 1.5 MHz. The occurrence of ultra-harmonic frequency components (1.5 f_0_, 2.5 f_0_, 3.5 f_0_) is a very sudden event (<256 µs), which can occur over the burst period.

Figure 2 shows an example of frequency responses from a microbubble cavitation signal measured at different times (before and after 3.3 ms) for the hemorrhagic case. The spectral analysis of successive temporal windows shows the occurrence of ultra-harmonic activity inside an ultrasound burst in less than 256 µs. In this case, the peak amplitude increased by 15 dB, 14 dB, and 10 dB for the 1^st^ (1.5 f_0_), the 2^nd^ (2.5 f_0_), and the 3^rd^ (3.5 f_0_) ultra-harmonic components, respectively. The presence of an initial low-amplitude peak at 1.5 f_0_ slightly masked the ultra-harmonic increase at this frequency, suggesting that this detection method would be more sensitive by including the 2^nd^ and the 3^rd^ ultra-harmonic components. Conversely, no increase in the noise level (broadband emission) was detected. Thus, the ultra-harmonic increase is not necessarily associated with inertial cavitation and can independently indicate the occurrence of adverse effects.

**Figure 3:**
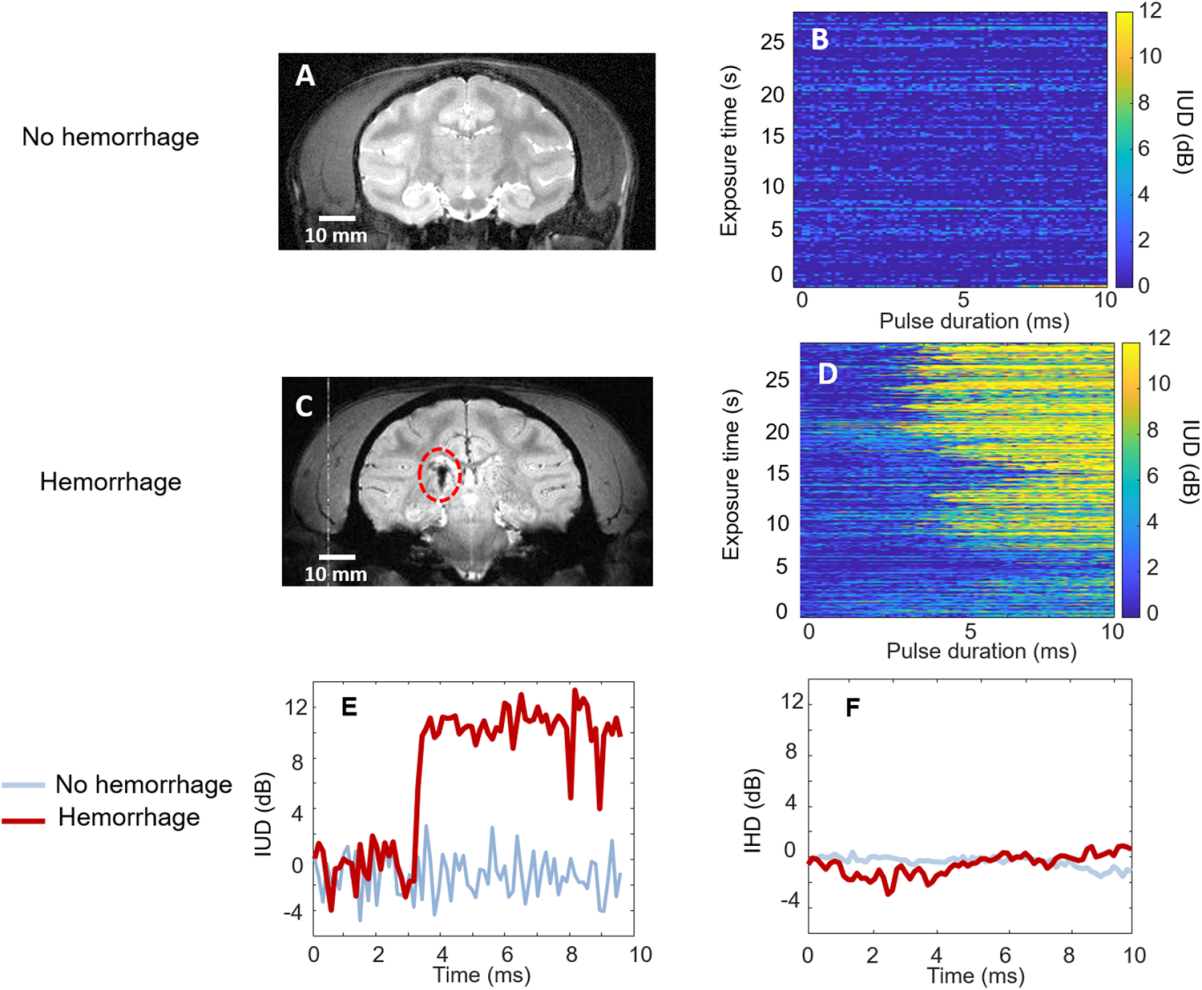
T2-weighted images in the focal region for safe (**A**) and hemorrhagic (**C**) 2 weeks after BBB opening in NHP. Evolution of IUD measured over ultrasound bursts (0.5 MHz, 10 ms) for safe (**B**) and hemorrhagic (**D**) BBB opening. Examples of IUD (**E**) and IHD measurements (**F**) for safe (blue) and hemorrhagic (red) BBB opening. Cavitation doses were normalized by a reference value corresponding to the ultra-harmonic cavitation dose from the 1^st^ window of 128 µs.

Examples of the evolution of the IUD indicator in NHP defined in section 2.2. are displayed in Fig. 3 for the sonications that resulted in safe (Fig. 3B) and hemorrhagic (Fig. 3D) BBB openings. Please note that the restricted memory of our recording device limited the PCD acquisitions for sessions 1 to 4. Thus, the IUD calculation was only performed on the first 27.5 seconds of the ultrasound procedure to allow the comparison between the 15 sessions. However, this data analysis is consistent with the period where most of the potential damages may occur. The occurrence of sudden ultra-harmonic emissions was associated with the presence of hemorrhages in the NHP brain, as shown in Fig. 3D. For these two examples, 30-s sonication resulted in sudden ultra-harmonic events (*i.e*., IUD > 8 dB) that occurred in 80% of the bursts for the hemorrhagic case and only in 0.7% of the bursts for the safe case. For all the safe BBB opening cases, the rate of occurrence of ultra-harmonic events was always lower than 4.0%. Interestingly, no successive repeated events (*i.e*., for two consecutive ultrasound bursts) were detected. For the hemorrhagic case, the generation of ultra-harmonics occurred at 5.40 ms ± 2.10 ms (standard deviation). In some cases (Table 2, session #4), a high occurrence of ultra-harmonic events in NHP can be potentially created due to the narrow safe pressure range caused by high variability of ultrasound transmission through the skull, as demonstrated by Kamimura *et al*. 2018^12^. These results suggest that a long pulse (at least one thousand cycles) is necessary to induce the instantaneous occurrence of ultra-harmonics and bubble destabilization at regular pressure levels used for BBB openings. Examples of the temporal evolution of ultra-harmonic cavitation inside a 10-ms burst are given in Fig. 3E for safe (blue) and hemorrhagic (red) BBB opening. The curve obtained for the hemorrhagic case can be decomposed in 3 stages. At the beginning of the burst (from 0 to 3.3 ms), no evolution of the ultra-harmonic level is observed. At this stage, the IUD is stable with a noise level between −4 and 4 dB. At 3.3 ms, the IUD increased by more than 10 dB. The increase at this stage occurred in less than 256 µs (corresponding to 2 temporal windows in our measurement). Then, in the last stage, the amplitude of ultra-harmonic oscillations reached a plateau and remained constant around 10 dB until the end of the burst. For the non-hemorrhagic case, the IUD did not exceed the noise level (±4 dB). By determining an adapted threshold value (for example, 8 dB), it was possible to discriminate the hemorrhagic case from the safe case by monitoring the temporal evolution of the ultra-harmonic emissions within a burst. Similar to IUD, the intrapulse harmonic dose (IHD) was calculated by considering the frequency signal at 2f_0_,3f_0_, and 4f_0_. No substantial change in the IHD was observed over the pulse duration, as shown in Fig. 3F. This result confirms that only the intrapulse monitoring of ultra-harmonic components (IUD) is a relevant indicator for damage prediction.

**Table 2:** Analysis of IUD events for NHP sessions.

Session #	Feedback loop based on ICD	% occurrence	Mean delay (ms)	Consecutive events	Lesion
1	NO	1.3	1.8	NO	NO
2	NO	0.7	3.2	NO	NO
3	NO	0.7	5.2	NO	NO
4	NO	80.0	5.4	YES	YES
5	YES	2.0	4.3	NO	NO
6	YES	0.0	—	NO	NO
7	YES	0.0	—	NO	NO
8	YES	1.3	0.5	NO	NO
9	YES	0.0	—	NO	NO
10	YES	0.0	—	NO	NO
11	YES	4.0	1.4	NO	NO
12	YES	0.0	-	NO	NO
13	YES	2.0	4.8	NO	NO
14	YES	3.4	3.8	NO	NO
15	YES	8.0	3.6	YES	SUSPECTED

**Figure 4:**
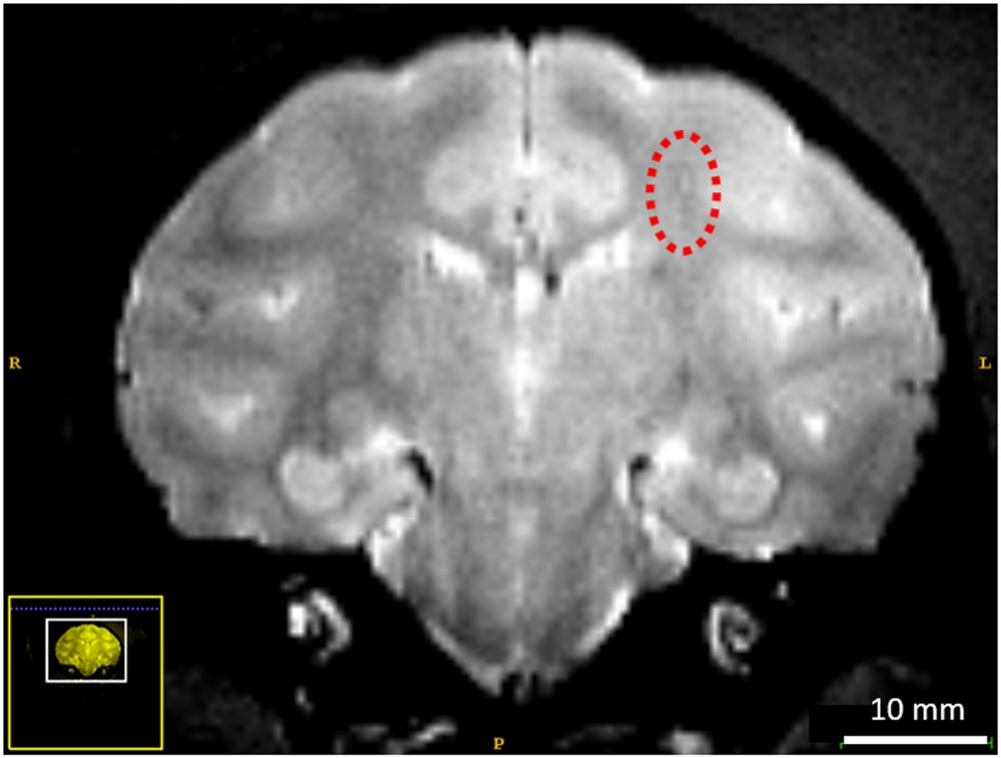
T2-weighted images one month after BBB opening shows a suspicious contrast in the focal region (dashed circle). No residual BBB leakage was detected.

Another BBB opening session resulted in an intermediate case showing a suspicious hyposignal in the sonicated area on late T_2_ images. IUD calculation showed an abrupt increase of IUD for a small portion of bursts (8.0% of the total), as illustrated in Fig. 4. Interestingly, multiple IUD events were detected for consecutive bursts. This case illustrates the sensitivity of the index that seems dose-dependent with the severity of bleeding. The results for NHP are summarized in Table 2.

### Observation of the ultra-harmonic cavitation on rat BBB opening

**Figure 5:**
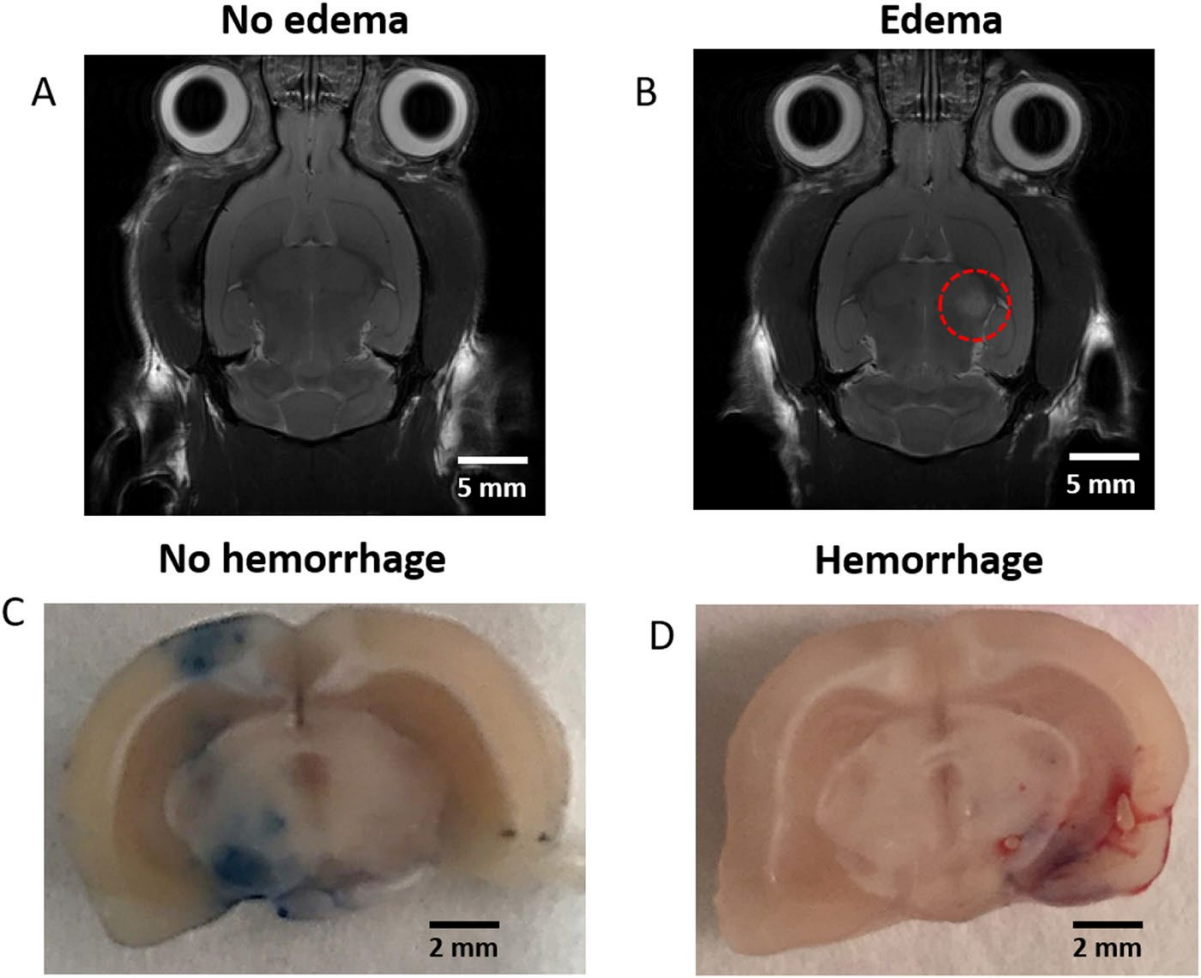
Representative safe and unsafe ultrasound-induced BBB opening in rats. A real-time feedback control based on the detection of inertial cavitation events set the sonications to safe levels. MRI sequences (T2-weighted, 7 T) identified the presence of post-opening edema. Rat brain image (**A**) without edema and (**B**) with edema (red circle) following sonication. Similarly, post-mortem analysis confirms the safety of ultrasound-induced BBB opening without apparent damages using Evans Blue dye (**C**) and with visible hemorrhages following sonication (**D**). These results show the need for more robust and more reliable controls.

Based on prior observations on NHPs, rat experiments allowed to study the robustness (interspecies using different setups) of this approach to the discrimination of safe and unsafe BBB opening. More critical cases were assessed in rats. In this protocol, the ultrasound-induced BBB opening was performed using the feedback control described in section 2.3. Despite this precaution, undesired effects such as edema and local hemorrhages were observed in some of the rats (Fig. 5). As shown in Fig. 5B for one rat, the presence of a hyperintense signal corresponding to edema (dashed circle) can be visualized on T_2_– weighted MRI. In line with these results, the post-mortem analysis revealed a localized hemorrhage in the targeted area (Fig. 5B). These data confirm that setting a threshold for inertial cavitation requires careful validation in different species before implementation. Detecting inertial cavitation using a conventional full-pulse analysis can be a late indicator as microbubbles are already at a risky oscillatory regime, potentially harmful for surrounding tissues. Moreover, averaging over a burst length tends to suppress transient cavitation signals, thus hindering the cavitation detection.

Quantitative results and observations obtained in 8 rats are summarized in Table 3. MRI and/or gross pathology confirmed brain damage in half of the rats, and the occurrence of damage is related to a high number of IUD events (>24). More interestingly, the repeated occurrence of this phenomenon (for at least 2 consecutive bursts) was always associated with the presence of undesirable effects (edema, hemorrhage). Furthermore, our results with NHPs suggest that the severity of brain damage was high (severe bleeding) as the number of occurrences increased. The mean time of IUD occurrence throughout the pulse was 3.15 ± 1.80 ms.

**Table 3:** Analysis of IUD events for rat sessions.

Rat ID	% MDD events	Mean delay (ms)	Consecutive events	Lesion
A	0.2	4.98	NO	NO
B	0.4	6.51	NO	NO
C	0.5	1.32	NO	NO
D	1.3	2.31	NO	NO
E	4.4	2.05	YES	LIGHT BLEEDING
F	5.5	4.91	YES	EDEMA
G	10.0	2.38	YES	SEVERE BLEEDING
H	10.4	3.43	YES	SEVERE BLEEDING

## Discussion

The differences in the ultrasound parameters applied for the two experiments can explain the difference in number and latency of IUD events observed in rats and NHP. Firstly, the transmission of ultrasound through the macaque skull requires a lower ultrasound frequency (500 kHz compared to 650 kHz for rats). Secondly, a recent study suggests that the excitation frequency plays a significant role in microbubble stability^31^. In this study, the authors demonstrate a relationship between the excitation frequency and the time delay necessary for the rupture of the microbubble shell that is different in both species. Furthermore, due to the feedback control loop based on a tolerable threshold of inertial cavitation, the acoustic pressure applied in rat and NHP protocols varies over sonication time.

Previous studies have introduced metrics for cavitation activity, where the intensity of bubble collapse could be measured by the level of broadband emission, the likelihood for a cavitation event measured by the cavitation probability quantification, and the duration of cavitation and relation with replenishment of cavitation nuclei determined by the cavitation persistence^18,32^. Here, the analysis of the segmented backscattered signal allowed to define the moment when the potential destabilization of microbubbles occurred, before bubble collapse with broadband emission. Our results indicate that ultra-harmonic content appeared when excitation burst exceeded a few milliseconds. By only considering mean delays when lesions are observed (other detected IUD events are considered as isolated with not relevant values), one can see that destabilization occurs after the transmission of at least 1330 cycles for rats (2.05 ms at 650 kHz) and 7200 cycles for NHP (3.60 ms at 500 kHz). The range of the time onset for consecutive events varied for NHP (between 3.6 ms and 5.4 ms) and rats (between 2.05 ms and 4.91 ms). This variability across species and interspecies could be attributed to the different ultrasound transmission through the skull from animal to animal. For example, in rats, the transmission factor through the skull in acoustic pressure amplitude can vary from 58% to 88% as a function of the body mass (from 200 g to 400 g) and the position of the transducer above the skull^33^. Therefore, it is desirable to adjust (or stop) the ultrasound sequence in real-time to ensure the safety of the procedure before harmful cavitation activity occurs. The sequence can be adapted in terms of amplitude and burst duration (number of cycles). Previous studies report an increase in the extent of BBB opening with an increase of the burst length^34,35^. However, it has been shown that very long bursts (> 10 ms) do not increase BBB opening further^2^, supporting the hypothesis that microbubble destabilization may occur during long burst duration^36^. Our results suggest that the number of cycles can be adjusted to values found in real-time to avoid microbubble destabilization and potential damages.

Beyond safety, the IUD parameter can be used to optimize the ultrasound sequence by determining the maximum tolerable burst length before microbubble destabilization. In terms of efficacy, being able to optimize burst length should maximize the bubble-vascular wall interaction for each burst as well as increase bubble lifetime in the circulation. This second phenomenon increases the useful sonication time and thus probably contributes to improved efficacy. The use of long bursts is also known to allow the reduction of the acoustic amplitude threshold necessary to induce BBB disruption^37^. This result is particularly interesting for clinical translation, where it can be challenging to reach a high power level due to the reduced ultrasound transmission through the human skull. Hynynen *et al*. reported that short pulses (10 µs, 2 kHz pulse repetition frequency) failed to produce BBB disruption below power levels that resulted in tissue damage^38^.

On the contrary, in a recent study, Morse *et al*. have suggested the use of multiple short bursts emitted at high pulse repetition frequency separated by off time in the range of microseconds to improve the safety of drug delivery^39^. The burst defined in this study was composed of 5-cycle short pulses (5 µs) repeated every 800 µs for 10 ms. The rapid short-pulse sequence (RaSP) was compared to a sequence composed of long-pulses of 10 ms for BBB disruption in mice. Using RaSP, the authors reported dextran delivery into the brain without ultrasound-associated damage. The BBB returned to its normal permeability within 10 minutes (compared to 4–48 hours for long-pulses). Thus, RaSP may be delivering lower amounts of drugs than long-pulse sequences due to the short duration of the permeability change in the BBB.

Ultra-harmonic (and probably subharmonic) emissions result from an instantaneous phenomenon (<256 µs) that may occur during an ultrasound burst. Once emitted, this frequency content persists until the end of the burst excitation (see Fig. 3D). Figure 3D also shows that when successive bursts are transmitted, the ultra-harmonic dose at the end of the acquisition returns to its initial level. This observation is explained by the off-time interval between two successive bursts used in our study (>90 ms) that allows the substitution of the destabilized microbubbles by a “fresh” microbubble cloud. Many hypotheses could explain the instantaneous occurrence of both sub and ultra-harmonic contents from lipid-shelled microbubbles. This specific emission signature has been demonstrated to be associated with the generation of microbubble non-spherical oscillations^27,40^. On the one hand, strongly asymmetric oscillations that generate sub and ultra-harmonics could result from the deflation of microbubbles^41^. Long sonication burst can induce diffusive gas loss^20^ and lipid material shedding from the encapsulating shell^22^. Although this hypothesis is conceivable, a variation of the frequency response over time is expected due to a progressive deflation of microbubbles during the burst, which appears to be in contradiction with our observation. Once an abrupt increase of the ultra-harmonic content was detected, the amplitude of ultra-harmonics remained stable until the end of the burst (Fig. 3E). On the other hand, non-spherical oscillations could also result from ultrasound-induced coalescence, as recently shown by Cleve *et al*.^42^. After coalescence, the resulting bubble has acoustic properties different from those of the original bubbles^25^. Coalescence must be controlled as violent phenomena induced by the oscillations of large microbubbles may result in substantial and undesirable local bioeffects. Finally, a particular regime of microbubble oscillations, characterized by period-doubling in the time-domain response, can be reached when microbubbles are excited near their inertial cavitation threshold^43^. In the frequency domain, this regime is the source of sub and ultra-harmonics. This hypothesis further supports our conclusions that these signatures can be used in feedback controllers to prevent inertial cavitation events, and consequently, potential damages in the brain.

Sudden variation in the frequency content can also be induced by the initial cavitation of bubble clusters confined at the focus^44^. This initial cavitation activity could result in an uncontrolled expansion of the BBB opening in the treated area. Responses from microbubbles located at different positions in the brain cannot be isolated using single-element PCD due to a lack of spatial resolution. Regardless of the hypothesis used to interpret the data, the safety indicator defined in our study shows that a sudden and repeated variation of ultra-harmonic content during the procedure is deleterious.

Our method has the added advantage of not requiring the acquisition of a baseline before microbubble injection. The reference is defined for each burst on the frequency analysis of the 1^st^ temporal window considered as the initial state of microbubbles. Besides a noticeable gain of time and memory, this approach also has the advantage of being insensitive to patient motion that could occur during sonication (a new reference is calculated at every pulse repetition frequency) or electrostatic effects from MRI that would alter PCD measurements between pre-acquisition and sonication. Furthermore, the direct comparison of microbubble responses to their initial state improves the robustness of the IUD indicator.

Conventional PCD analysis that calculates indexes at every burst is equivalent to having an overview of the frequency content contained in the echo. Both sub and ultra-harmonic components can be diluted in the frequency response, primarily when the destabilization occurs at the very end of the burst. Hence, we expect that our methodology monitoring the temporal evolution of the frequency content inside the burst would be more sensitive than global peak detection.

The examples given in this study (NHP and rats) are restricted to the tracking of ultra-harmonic frequencies only. The receive transducers used for cavitation detection were centered at three times the transmit frequency (f_0_), and their limited bandwidth did not allow a reliable measure of the subharmonic component. However, we believe that the use of a suitable transducer for subharmonic detection would allow the observation of similar behavior at f_0_/2. The combined exploitation of several ultra-harmonic components (3f_0_/2, 5f_0_/2, 7f_0_/2) and a subharmonic component (f_0_/2) will further improve the sensitivity of the approach. Thus, we suggest the use of a transducer centered at 3f_0_/2 for PCD to optimize the recovery of the frequency content ranging from f_0_/2 to 5f_0_/2. It would be interesting to evaluate the potential of a wideband transducer, such as CMUT, for the detection of these nonlinear cavitation events^45^. The broadband emission can still be employed as an endpoint criterion.

In this retrospective data processing, we could observe in multiple experiments with rats and NHPs a consistent destabilization of the microbubbles that was evident in cases that resulted in hemorrhage and edema. An implementation where we could stop the sonication when destabilization is detected would provide additional information on the bubble destabilization and occurrence of damages. However, real-time monitoring requires ultrafast computation (in the order of hundreds of µs) for the temporal windowing, the fast Fourier transform, and the calculation of cavitation doses. Recently, Cornu *et al*. have used an FPGA-based feedback loop to track subharmonic emissions from microbubbles^46^. The authors successfully monitored every 250 µs the subharmonic level emitted during long-pulsed sonication (50 ms), showing that ultrafast feedback control of the emission is feasible. In this case, the loop was used to ensure a steady level of subharmonic emission during the sonication. In future studies, we will explore the implementation of our algorithm in FPGAs. This implementation could potentially be used to optimize the pulse sequence, such as the pulse length could be reduced each time consecutive IUD events are detected. The optimized sequence could provide a safer range of pulse duration while keeping the microbubble activity as high as needed for efficient BBB disruption.

The IUD indicator defined here can be implemented in a feedback loop and combined with other conventional strategies such as the quantification of the inertial cavitation dose to ensure the safety of therapeutic ultrasound interventions. Moreover, our results suggest that correlated occurrence of IUD events (two consecutive events) could be a potential predictor of brain damage, as some isolated IUD events can be detected due to a wrong calculation related to the difficulty in measuring harmonic contents through a skull (lack of sensitivity and noise). Therefore, it is possible to define an acceptable number of non-consecutive destabilization events before changing ultrasound settings to overcome the potential limitations of the data acquisition quality.

Optimal parameters for post-processing vary as a function of the ultrasound sequence. For example, the number of windows and their size directly depend on the length and the frequency of the ultrasound burst used for therapy. Efficient reversible BBB opening is achieved for ultrasound burst length varying from a few tens of microseconds to several hundreds of milliseconds^37^. From a theoretical point of view, two temporal windows having equivalent size are sufficient to observe the impact of bubble destabilization on the frequency response. However, this implementation would be inadequate in terms of time accuracy and sensitivity to adverse events. Thus, it is recommended to subdivide the backscattered signal into multiple consecutive temporal windows, relatively short (a few tens of cycles), to enhance the sensitivity of the approach. The number of cycles (*n*_*c*_) contained in the temporal window must be high enough (at least eight cycles) to avoid frequency overlapping during spectral analysis. Spectral bands (*Δf*) selected to identify and quantify frequency components depend on the transmit frequency f_0_ and the sampling frequency of the measurement device. Our results indicate that signal processing is suitable for spectral bands *Δf* ranging from 0.04 f_0_ to 0.5 f_0_. The spectral bandwidth must be adapted to the number of cycles of the temporal window to reduce the impact of noise (if *n*_*c*_ is high, *Δf* is small and conversely).

## Conclusion

Our results show the occurrence of an instantaneous nonlinear phenomenon, characterized by the generation of ultra-harmonics after a few milliseconds of sonication. The intra-pulse monitoring made it possible to observe this specific oscillation regime attributed to microbubble destabilization and related to the occurrence of unwanted side effects (e.g., hemorrhage, edema). A new indicator based on the intrapulse detection of ultra-harmonic events has been defined to discriminate potentially dangerous from safe ultrasound-induced BBB opening conditions. This safety parameter is useful, particularly for monitoring the BBB opening in primates, where transmitted pressure through the skull is difficult to predict.
